# Quality assurance for deep learning-based proton therapy planning in routine clinical use for oropharyngeal cancer

**DOI:** 10.1016/j.phro.2026.101067

**Published:** 2026-08-20

**Authors:** Ilse G. van Bruggen, Minke J. Brinkman-Akker, Ilse D. Jonkhof, Johannes A. Langendijk, Stefan Both, Erik W. Korevaar

**Affiliations:** Department of Radiation Oncology, University Medical Center Groningen, Groningen, the Netherlands

**Keywords:** artificial intelligence, deep learning, automated treatment planning, robust optimization, intensity modulated proton therapy, head and neck cancer

## Abstract

Quality assurance is required to ensure safe and reliable use of deep learning (DL)–based intensity modulated proton therapy (IMPT) planning for oropharyngeal cancer patients. This study presents a range of quality assurance measures applied during routine clinical use and include manual adjustments to DL-based plans, independent organ-of-interest dose guidance and multidisciplinary plan review. DL-based plans were clinically acceptable for all 78 patients and achieved 1.1 Gy (RBE) lower parotid dose than manual plans. The implemented quality assurance measures enabled safe routine clinical use of DL-based IMPT planning over 28-months.

## Highlights

• Quality assurance supported safe clinical use of deep learning-based planning.
• All deep learning-based plans were clinically acceptable for 78 patients.
• Deep learning-based plans achieved 1.1 Gy (RBE) lower parotid dose than manual plans.

## Graphical abstract

**Figure:**
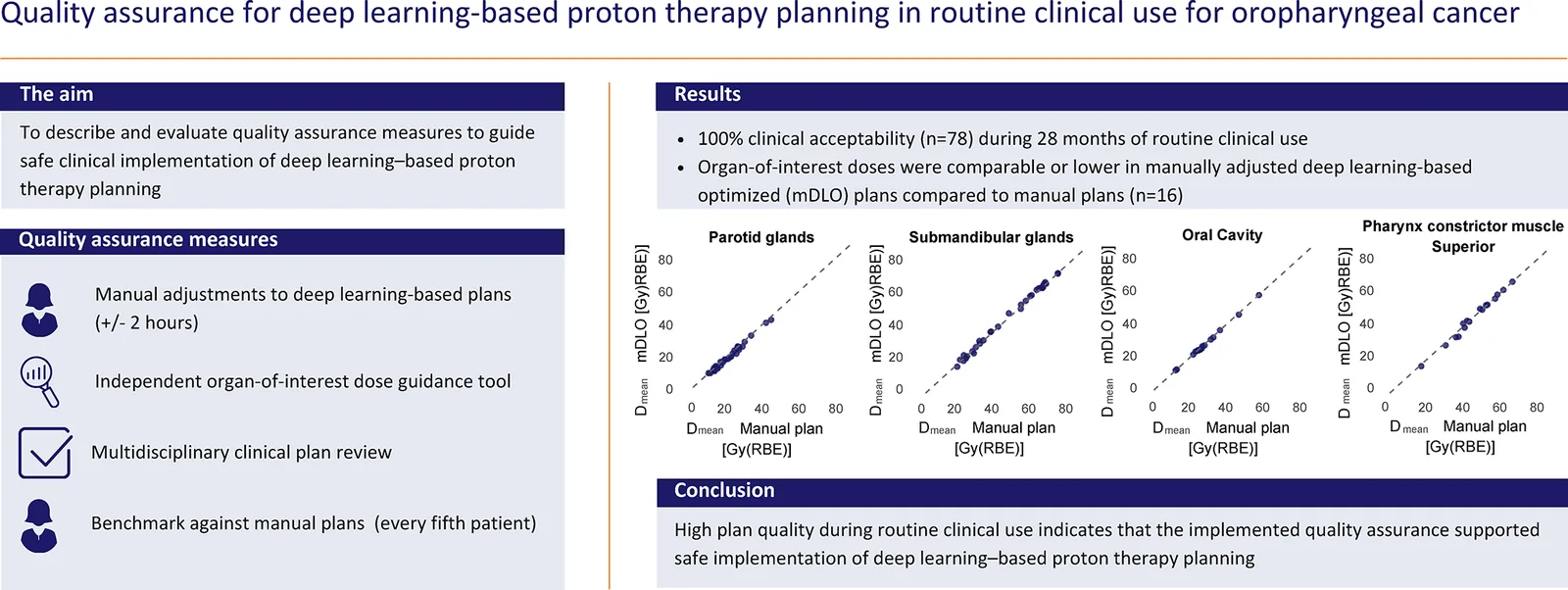

## 1 Introduction

Manual radiotherapy planning is both time-consuming and resource-intensive, often requiring hours or even days to complete. This is particularly true for intensity modulated proton therapy (IMPT) when setup and density uncertainties need to be considered to ensure robust target coverage [1]. Additionally, variability in skills and experience of treatment planners may influence plan quality [2], [3], [4]. Automated treatment planning using artificial intelligence (AI) models has the potential to enhance IMPT workflows by improving efficiency and consistency [5], [6], [7], [8], [9], [10].

Several studies have shown potential in the implementation of AI-based planning into clinical practice [11], [12], [13]. McIntosh et al.*,* reported that 86% of automatically generated photon plans for prostate cancer were clinically acceptable, indicating clinical feasibility of AI-based planning in a prospective setting [11]. Furthermore, 86% of AI-based volumetric modulated arc therapy (VMAT) plans were clinically acceptable after manual adjustments in a prospective study on breast cancer patients [12]. Similarly, a previous prospective study on deep learning (DL)-based IMPT planning for oropharyngeal cancer (OPC) reported a clinical acceptability rate of 87% [13].

While prospective studies on AI-based treatment planning have reported promising clinical results, translation to routine clinical use introduces new challenges [14], [15], [16]. In study settings, plan quality is commonly assessed through comparison with manually generated reference plans. However, following clinical deployment of AI-based planning, such manual reference plans are no longer available for each patient. In addition, the iterative planner adjustments that traditionally guide the achievable balance between target coverage and organ-of-interest (OOI) sparing become less explicit in semi- or fully automated workflows. As a result, it is more difficult to determine whether OOI doses are close to the optimal achievable level for an individual patient using conventional dose–volume metrics alone [17].

Consequently, plan quality assurance is required to ensure that AI-based plans meet clinical standards for individual patients in routine clinical use. The aim of this study was to assess plan quality of DL-based IMPT planning in routine clinical use, and to describe the implemented quality assurance measures to ensure safe implementation.

## 2 Materials and methods

### 2.1 Patients

All patients with OPC planned for radiotherapy with curative intent with a prescribed dose of 70 Gy (RBE),^1^ assuming a constant relative biological effectiveness (RBE) of 1.1 for protons, to the therapeutic clinical target volume (CTV_therapeutic_) and 54.25 Gy (RBE) to the bilateral elective lymph node areas (CTV_elective_) were eligible for DL-based IMPT planning. All patients provided written informed consent to participate in a standardized follow-up program, which was approved by the Medical Ethics Review board of the University Medical Center Groningen (NCT02435576).

Between March 2023 and June 2025 (28 months), a total of 99 patients were considered for DL-based planning. Of these, 21 patients were excluded due to the absence of a DL-trained treatment planner (*n* = 5), lack of a DL license (*n* = 2) and missing data-sharing approval (*n* = 10). In four patients, a fully manual plan was generated because of specific anatomical challenges, such as the presence of dental fillings.

The final study cohort consisted of 78 patients treated with DL-based IMPT. Of these patients, 51 were planned using RayStation 11B and 27 using RayStation 2023B (RaySearch Laboratories AB, Stockholm, Sweden). In addition, a benchmark cohort of 16 patients (every fifth patient treated) was selected, for whom a fully manual plan was generated by a treatment planner for comparative evaluation.

### 2.2 Clinical treatment planning workflow

The clinical workflow for generating DL-based plans is described below, while detailed descriptions of beam configuration, computational hardware and treatment delivery parameters are provided in Supplementary Material A. For new patients, the workflow involved the following steps. A three dimensional (3D) U-Net architecture was used to predict a voxel-wise dose distribution based on target and OOI contours [19]. The predicted dose distribution was then used as input for a robust mimicking optimization process, in which a conventional IMPT optimization was performed to generate a machine deliverable plan that closely reproduced the predicted dose while explicitly accounting for setup and range uncertainties. Robust optimization took approximately 30 min and included 21 scenarios for the CTV using 3 mm isotropic uncertainty and 3% density uncertainty in RayStation 11B and 2023B [20], [21]. Dose calculations were performed using a Monte Carlo algorithm with a 3 mm dose grid and a statistical uncertainty of 0.5% per beam. Hereafter, plans generated with DL-based planning are referred to as deep learning–based optimization (DLO) plans.

A treatment planner subsequently manually adjusted the standard DLO (sDLO) plan by adding objectives or adjusting objective weights. This refinement, which required approximately two hours, resulted in the manually adjusted DLO (mDLO) plan and served as a quality assurance step to determine whether the plan could be further improved and to test that as low as reasonably achievable (ALARA) was reached for OOI doses in individual patients. Furthermore, an independent OOI dose guidance tool was used to assist the treatment planner in determining feasible OOI doses [22]. Doses to OOIs in the mDLO plan were aimed to be equal or lower than the OOI doses predicted by the tool. The tool used linear regression models, with OOI volume percentages overlapping with target volumes as predictors.

Finally, the mDLO plan was evaluated in a multidisciplinary meeting involving a radiation oncologist, treatment planner and medical physicist by a slice-by-slice visual inspection and evaluation of clinical goals, dose parameters and normal tissue complication probabilities (NTCPs) [23].

### 2.3 DL-based IMPT planning configuration

The current study used the same training set of 60 patients as previously described [13]. Prior to the start of this study, prediction and mimicking parameters of the DL-based planning workflow were updated. Specifically, coverage of CTV_elective_ was improved and the glottic area, thyroid and cervical esophagus were added, as these OOIs were not included in the original configuration. After 14 months, the treatment planning system (TPS) was upgraded to RayStation 2023B and the prediction and mimicking parameters were updated to ensure compatibility with the new TPS version. The prediction and mimicking parameters are provided in Tables S1-S3 of the Supplementary Material.

### 2.4 Plan quality evaluation and statistical analysis

The plan quality assurance measures applied in routine clinical practice are summarized in Table 1.

**Table 1:** **Quality assurance measures for DL-based IMPT planning.** Clinical quality assurance methods were applied during routine practice, while their effectiveness was evaluated retrospectively at the cohort level in this study.

Quality assurance measure	Evaluation in this study
Manual refinement of sDLO to mDLO plans	Comparison of sDLO and mDLO plans with respect to predefined plan quality criteria⁎ across all patients (*n* = 78)
Independent OOI dose guidance tool	Comparison of independently predicted mean OOI doses with corresponding mDLO plan doses to assess OOI dose sparing. Agreement was quantified using Pearson correlation and linear regression across all patients (n = 78)
Multidisciplinary review ⁎⁎ of mDLO plans	Clinical review of plan acceptability and achievement of predefined plan quality criteria* for individual patients across the full cohort (n = 78)
Benchmark mDLO plans against blinded manual plans (for every fifth patient)	Comparison of mDLO and manual plans to predefined plan quality criteria* in a benchmark cohort (*n* = 16)

Abbreviations: sDLO plan: standard deep learning optimization plan, mDLO plan: manually adjusted deep learning optimization plan, OOI: organ-of-interest, CTV: clinical target volume

⁎ Plan quality evaluation criteria included assessment of robust CTV coverage and normal tissue sparing. Robust CTV coverage was evaluated using voxel-wise minimum and maximum dose distributions derived from 28 perturbed scenarios [24], requiring a CTV D98% ≥ 94% and voxel-wise maximum doses < 78 Gy (RBE). In addition, OOI dose metrics and normal tissue complication probabilities for grade ≥ 2 and ≥ 3 dysphagia and xerostomia were evaluated.

⁎⁎ Multidisciplinary review was performed by a radiation oncologist, treatment planner, and medical physicist and included slice-by-slice visual inspection of dose distributions and quantitative plan quality evaluation as described above.

These measures were evaluated for all patients to assess whether they were sufficient to ensure consistent plan quality across patients and during clinical use.

Statistical analysis was performed to identify any significant differences between the plans using a Bonferroni-corrected Wilcoxon signed-rank test. Bonferroni-corrected *p*-values were used to account for multiple comparisons. A *p*-value lower than *p* = 0.013 (α = 0.05/4) for target parameters, *p* = 0.017 (α = 0.05/3) for serial OOIs, *p* = 0.006 (α = 0.05/8) for parallel OOIs and *p* = 0.013 (α = 0.05/4) NTCPs was considered statistically significant.

## 3 Results

**Fig. 1:**
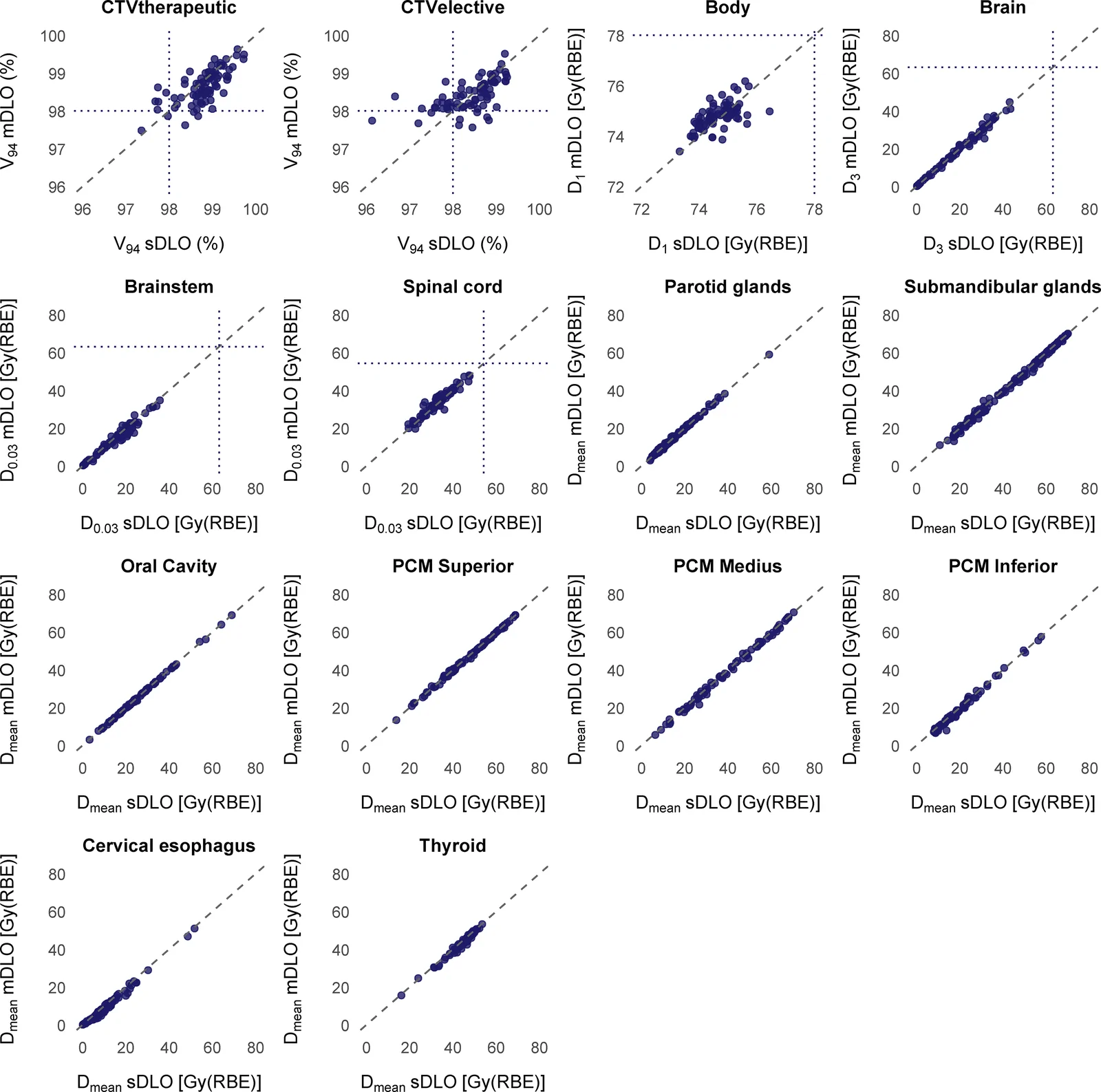
Target parameters and organ-of-interest doses for sDLO and mDLO plans (*n* = 78). The values are plotted as a blue dot for each patient, the dashed grey line represents the identity line (x = y). sDLO: standard deep learning optimization, mDLO: manually adjusted deep learning optimization. (For interpretation of the references to colour in this figure legend, the reader is referred to the web version of this article.)

Manual adjustments resulted in an increase of clinical goal achievement from 91% (sDLO, *n* = 71) to 93.6% (mDLO, *n* = 73) in CTV_therapeutic_ and 78.2% (sDLO, *n* = 61) to 88.5% (mDLO, *n* = 69) in CTV_elective_ (Fig. 1, Supplementary Table S4). All other clinical goals were met in both the sDLO and mDLO plans (Supplementary Table S4). All NTCP values were significantly reduced in the mDLO plans; grade ≥  2 dysphagia (sDLO = 15.9%, mDLO = 15.5%), grade ≥  3 dysphagia (sDLO = 3.8%, mDLO = 3.7%), grade ≥ 2 xerostomia (sDLO = 37.9%, mDLO = 37.6%), grade ≥ 3 xerostomia (sDLO = 10.6%, mDLO = 10.5%, all *p* < 0.001) (Supplementary Table S5).

A very strong correlation was observed between the predicted mean dose by the independent dose prediction tool and the doses in the mDLO plan for all OOIs (Pearson *r* = 0.96–1.00, *P* < 0.001) (Supplementary Table S6, Fig. S1). The slopes of linear fits through the origin ranged from 0.83 to 0.98.

All 78 mDLO plans were considered to be clinically acceptable and used for IMPT treatment. All clinical goals were met in 68 patients. In the remaining 10 patients, the V_94%_ values for CTV_therapeutic_ and/or CTV_elective_ were below the threshold of 98% (97.5% to 97.9%).

All clinical goals were met in manual plans for all patients (*n* = 16) in the benchmark cohort, while mDLO plans reached all clinical goals in 14/16 patients (Supplementary Table S4). Dose to the spinal cord and parotid glands were significantly lower in the mDLO plans compared to the manual plans (spinal cord mDLO: D_0_ = 30.3 Gy (RBE), manual: D_0_ = 33.2 Gy (RBE), *p* = 0.004; parotid glands mDLO: D_mean_ = 15.8 Gy (RBE), manual: D_mean_ = 16.9 Gy (RBE), *p* = 0.002) (Supplementary Table S7, Fig. S2). No other significant differences were found between the mDLO and manual plans. Supplementary Fig. S3 shows an example sDLO, mDLO and manual plan.

## 4 Discussion

In this study, clinically integrated DL-based IMPT planning consistently produced clinically acceptable treatment plans for all OPC patients. All mDLO plans (*n* = 78) met the clinical acceptability criteria, and plan quality remained comparable to that of manual reference plans in the benchmark cohort (*n* = 16). These findings indicate that high-quality treatment plans can be achieved using DL-based IMPT planning in routine clinical practice.

Manual adjustments to sDLO plans to generate the final mDLO plans increased the proportion of patients meeting all clinical goals. This refinement allowed treatment planners to make patient-specific improvements. Although NTCP values decreased significantly after refinement, the average magnitude of these reductions was small and likely not clinically relevant. These findings suggest that sDLO plans could be sufficient for a substantial proportion of patients, while manual refinement remains available when additional adjustments are indicated.

An independent OOI dose guidance tool was used to provide an additional reference for expected dose values. The observed agreement between predicted and achieved OOI doses indicated that the tool can support interpretation of plan quality and help identify cases where further refinement may be considered, consistent with recommended quality assurance approaches of AI-based planning in radiotherapy [14], [15], [16].

In routine clinical use of DL-based IMPT planning, multidisciplinary slice-by-slice review of mDLO plans remained the primary quality assurance step. A high acceptance rate of mDLO plans (100%, *n* = 78) was observed in this study, which is in accordance with the results described by Bakx et al. [12]. They reported clinically acceptable plan quality in 86% of manually adjusted AI-based VMAT breast cancer plans. A clinical acceptability rate of 86% was also reported by McIntosh et al. for AI-based photon prostate cancer plans [11]. These results are consistent with previous evaluations of the current DL-based IMPT method for oropharyngeal cancer, where retrospective and prospective studies found clinical acceptability in 10 out of 10 and 13 out of 15 cases, respectively [13].

The benchmark cohort (for every fifth patient) served as an independent clinical reference to evaluate plan quality during routine use of DL-based IMPT planning. Comparison with blinded manual plans showed comparable target coverage and OOI sparing, providing reassurance that DL-based plans aligned with conventional planning standards. As clinical experience and trust with DL-based IMPT planning increases, the frequency of manual benchmark generation could be further reduced, for example, to every tenth patient.

A strength of this study is the evaluation of a DL-based IMPT planning in a real-world setting. However, the study has several limitations that should be acknowledged. First, the study was conducted in a single clinical setting and focused solely on oropharyngeal cancer patients. This limits the generalizability of the findings. External validation would be recommended to confirm the method's robustness and adaptability in diverse clinical environments. Second, the time spent on manual adjustments of DLO plans was not recorded, which limited the ability to quantify workflow efficiency gains. Nonetheless, a substantial time reduction is anticipated, as manual adjustments took approximately two hours, compared to the one to two-day duration required for full manual planning.

In conclusion, this study demonstrated that clinically integrated deep learning–based IMPT planning consistently produced high-quality, clinically acceptable plans for all 78 patients, with plan quality maintained during 28 months of routine clinical use. The applied quality assurance measures enabled safe implementation without the need for manual reference plans for each patient.

## Declaration of generative AI and AI-assisted technologies in the manuscript preparation process.

During the preparation of this work the authors used Microsoft Copilot in order to enhance language and readability. After using this tool, the authors reviewed and edited the content as needed and take full responsibility for the content of the published article.

## CRediT authorship contribution statement

**Ilse G. van Bruggen:** Writing – review & editing, Writing – original draft, Visualization, Software, Project administration, Methodology, Investigation, Formal analysis, Data curation, Conceptualization. **Minke J. Brinkman-Akker:** Writing – review & editing, Data curation. **Ilse D. Jonkhof:** Writing – review & editing, Methodology, Data curation. **Johannes A. Langendijk:** Writing – review & editing, Supervision. **Stefan Both:** Writing – review & editing, Supervision, Conceptualization. **Erik W. Korevaar:** Writing – review & editing, Supervision, Methodology, Conceptualization.

## Declaration of competing interest

The authors declare that they have no known competing financial interests or personal relationships that could have appeared to influence the work reported in this paper.

## Footnotes

1 Dose values are reported as Gy (RBE) according to the ICRU 93 definition [18]
