# Supplementary material for: Quality assurance for deep learning-based proton therapy planning in routine clinical use for oropharyngeal cancer
Source: Phys Imaging Radiat Oncol. 2026 Aug 20;41:101067. doi: 10.1016/j.phro.2026.101067 (PMC13571721; doi:10.1016/j.phro.2026.101067)
Supplement: Supplementary file 1 [file mmc1.pdf]

Table S1 Overview of prediction post-processing parameters of the deep learning-based planning method in RayStation 11B and 2023B. Specifications of all parameters are outlined in the "RayStation 11B – Scripting environments for Machine Learning" guide page 9-20.

**GoalType**	**Function**	**Value [Gy (RBE)]**	**ROI**
Target prediction settings			
MinDose	Aggressive	69.5	CTV70
MinDose	Aggressive	53.8	CTV54.25
MaxDose	Aggressive	71.0	CTV70+10mm
MaxDose	Aggressive	69.0	PTV54.25-CTV70
MaxDose	Aggressive	68.0	PTV54.25-PTV70
MaxDose	Aggressive	66.5	(CTV70+10mm)-PTV70
MaxDose	Aggressive	55.0	PTV54.25-(CTV70+15mm)
MaxDose	WeightedDvhshift	69.5 threshold 0.5	CTV70
OOI prediction settings			
MaxDose	Aggressive	62.0	PCMs/(CTV54.25+5mm)-(CTV70+10mm)
MaxDose	Aggressive	60.0	Cerebrum, Cerebellum, Brainstem, Cochlea_L, Cochlea_R
MaxDose	Aggressive	54.3	Parotid_L/(CTV54.25+5mm)-(CTV70+10mm), Parotid_R/(CTV54.25+5mm)-(CTV70+10mm), Submandibular_L/(CTV54.25+5mm)-(CTV70+10mm), Submandibular_R/(CTV54.25+5mm)-(CTV70+10mm), OralCavity/(CTV54.25+5mm)-(CTV70+10mm), PCMs/(CTV54.25+5mm)-(CTV70+10mm), GlotticArea/(CTV54.25+5mm)-(CTV70+10mm) Crico/(CTV54.25+5mm)-(CTV70+10mm), Esophagus_Cerv/(CTV54.25+5mm)-(CTV70+10mm), Thyroid/(CTV54.25+5mm)-(CTV70+10mm), Supraglottic/(CTV54.25+5mm)-(CTV70+10mm),
MaxDose	Aggressive	52.0	PCM_Inf/PTV54.25-CTV54.25, PCM_Med/PTV54.25-CTV54.25, PCM_Sup/PTV54.25-CTV54.25,
MaxDose	Aggressive	50.0	SpinalCord
MaxDose	Aggressive	40.0	Parotid_L_Out/(CTV54.25+8mm), Parotid_R_Out/(CTV54.25+8mm), Submandibular_L_Out/(CTV54.25+8mm), Submandibular_R_Out/(CTV54.25+8mm), PCM_Inf_Out/(CTV54.25+8mm), PCM_Med_Out/(CTV54.25+8mm), PCM_Sup_Out/(CTV54.25+8mm),
MaxDose	WeightedDvhShift	26.0 threshold 0.5	Parotid_L_Out, Parotid_R_Out Submandibular_L_Out, Submandibular_R_Out PCM_Inf_Out, PCM_Med_Out, PCM_Sup_Out
ReduceDose	Reduction	0.175	PCM_Inf_Out-(CTV54.25+15mm)
ReduceDose	Reduction	0.175	PCM_Inf_Out-(CTV54.25+5mm)
ReduceDose	Reduction	0.175	PCM_Inf-PTV54.25, PCMs-(CTV+15mm)
ReduceDose	Reduction	0.15	PCM_Med_Out-(CTV54.25+15mm), PCM_Sup_Out-(CTV54.25+15mm),
ReduceDose	Reduction	0.15	PCM_Med_Out-(CTV54.25+5mm) PCM_Sup_Out-(CTV54.25+5mm)
ReduceDose	Reduction	0.125	Parotid_L_Out-(CTV54.25+15mm) , Parotid_R_Out-(CTV54.25+15mm)
ReduceDose	Reduction	0.1	PCM_Med-PTV54.25, PCMs-(CTV+15mm)

ReduceDose	Reduction	0.1	Parotid_L-(CTV54.25+5mm), Parotid_R-(CTV54.25+5mm)
ReduceDose	Reduction	0.075	PCM_Sup-PTV54.25, PCMs-(CTV+15mm)

Abbreviations: CTV: clinical target volume, PTV: planning target volume, PCM: pharynx constrictor muscle, Sup: superior, Med: medius, Inf: inferior, L: left, R: right
The ‘out’ part refers to structures obtained by subtracting an OOI from the PTV expanded by 5mm, typically used to reduce the dose in the OOI without losing target coverage. Please note that the PTV in these settings is not used as a planning target volume, but only as a 3mm expansion of the CTV.
Symbols:
"/" denotes intersection
"-" denotes exclusion
"&" denotes union

Table S2 Overview of mimicking optimization functions of the deep learning-based planning method in RayStation 11B and 2023B. Specifications of all parameters are outlined in the “RayStation 11B – Scripting environments for Machine Learning” guide page 9-20.

**Function**	**Weight**	**Isoweights**	**Isodose**	**ROI**
MinRefDose robust	0			CTV70
MinRefDose	0			CTV54.25
MaxRefDose robust	0	10, 6, 1, 1	104, 100, 98, 0	CTV70
MaxRefDose	0	1, 1, 1, 0.1	104, 100, 77.5, 0	CTV54.25
MaxRefDose	5.8			PCMs-(CTV+15mm)
MaxRefDose	1.8			GlotticArea_Out, Crico_Out, (Esophagus_Cerv_Out)+3mm, Thyroid_Out, Supgraglottic_Out PCM_Inf_Out, PCM_Med_Out, PCM_Sup_Out
MaxRefDose	1.7			Parotid_R_Out, Parotid_L_Out, Submandibular_R_Out, Submandibular_L_Out OralCavity_Ext_Out
MaxRefDose	0.9			Parotid_L-PTV, Parotid_R-PTV, Submandibular_L-PTV, Submandibular_R-PTV, OralCavity_Ext-PTV, GlotticArea-PTV, Crico-PTV, (Esophagus_Cerv+3mm)-PTV, Thyroid-PTV, Supgraglottic-PTV
MaxRefDose	0.8			Cochlea_L, Cochlea_R, PCM_Inf-PTV, PCM_Med-PTV, PCM_Sup-PTV
MaxRefDose	0.2			SpinalCord
MaxRefDose	0.1			Brainstem
MaxRefDose	0.02			Body-(PTV+9mm)
MaxRefDose	0.01			Cerebrum, Cerebellum

Abbreviations: similar as in table S1

Table S3 Overview of additional objective functions applied during the mimicking phase of deep learning-based planning in RayStation 11B and 2023B. Specifications of all parameters are outlined in the "RayStation 11B – Scripting environments for Machine Learning" guide page 9-20.

**Function**	**Doselevel [Gy (RBE]**	**Weight**	**ROI**
MinDose robust	70.0	750	CTV70
MinDose robust	68.3	5000	CTV70
MinDose robust	67.2	5000	Ring3mmCTV70
MinDose robust	66.5	1	CTV70
MinDose robust	66.0	3000	ring3mmCTV70
MinDose robust	54.3	3500	CTV54.25
MinDose robust	52.9	7000	CTV54.25
MinDose robust	52.0	7000	ring3mmCTV54.25
MinDose robust	51.5	1	CTV54.25
MinDose robust	51.0	3000	ring3mmCTV54.25
MinDose	51.0	2500	CTV54.25
MaxDose robust	71.0	5000	CTV70+10mm
MaxDose robust	71.0	9000	CTV70+10mm
MaxDose robust	70.0	500	CTV70
MaxDose robust	57.0	7000	(CTV54.25+10mm)-(CTV70+10mm)
MaxDose robust	57.0	9000	(CTV54.25+10mm)-(CTV70+10mm)
MaxDose robust	54.3	150	CTV54.25-(CTV70+10mm)
MaxDose	71.0	5000	CTV70+10mm
MaxDose	71.0	5000	CTV70+10mm
MaxDose	57.0	8000	(CTV54.25+10mm)-(CTV70+10mm)
MaxDose	57.0	7000	(CTV54.25+10mm)-(CTV70+10mm)
UniformDose	70.0	500	CTV70
UniformDose	54.3	250	CTV54.25-(CTV70+10mm)

Abbreviations: CTV: clinical target volume, ring3mmCTV refers to a 3mm ring within the CTV, typically used to increase target coverage at CTV's perimeter

Supplementary Material A: Technical details of the clinical workflow for deep learning-based proton therapy planning

Beam configuration and avoidance structures

A class-solution beam setup was manually created for each patient. In the majority of patients (59%), this setup consisted of five coplanar beams with fixed gantry directions: two anterior oblique, two posterior oblique, and one lateral beam. Four beams were used in 9% of the patients, six beams in 24%, and seven beams in 8%, depending on individual anatomical considerations.

A 4 cm range shifter was incorporated in the lateral and two anterior oblique beams. To prevent beam delivery through regions with uncertain anatomy or variable tissue composition, avoidance structures (blocks) were placed in the shoulders, sinuses and, if applicable, dental fillings.

The initial beam configuration, including beam angles, range shifter usage, and avoidance structures was evaluated in a multidisciplinary meeting involving a radiation oncologist, treatment planner, and medical physicist prior to deep learning-based plan generation.

Computational hardware and treatment delivery parameters

Deep learning-based plan generation and optimization were performed using the following processors: Intel Xeon CPU Gold 6248R 3.00 GHz, Intel Xeon CPU E5-2698 v4 2.20 GHz, and Intel Xeon CPU Gold 6136 3.00 GHz, along with the Nvidia Quadro RTX 8000 GPU.

All intensity modulated proton therapy plans were optimized for the IBA ProteusPlus proton gantry (Ion Beam Applications SA, Louvain-la-Neuve, Belgium), using energy levels between 70 and 230 MeV. The corresponding spot sizes in air ranged from 6.5 mm to 3.0 mm (one sigma) and the minimum monitor units per spot was 0.02. The energy layer spacing was set to 'automatic with scale 1.0' and spot spacing to 'automatic with scale 0.8'.

Table S4 Achieved clinical goals for sDLO and mDLO plans for all patients (n=78) and mDLO and manual plans for the benchmark cohort (n=16). Results are shown in percentage of the achieved clinical goals.

	**All patients (n=78)**		**Benchmark cohort (n=16)**	
**Clinical goal**	**sDLO**	**mDLO**	**mDLO**	**Manual**
$CTV_{therapeutic}$ $V_{94\%}$> $D_{98\%}$ voxmin	71 (91%)	73 (93.6%)	14 (87.5%)	16 (100%)
Body D_1< 78 Gy (RBE) voxmax	78 (100%)	78 (100%)	16 (100%)	16 (100%)
$CTV_{elective}$ $V_{94\%}$> D98 voxmin	61 (78.2%)	69 (88.5%)	14 (87.5%)	16 (100%)
Brain D_3< 63.1 Gy (RBE)	78 (100%)	78 (100%)	16 (100%)	16 (100%)
Brainstem $D_{0.03}$< 63.1 Gy (RBE)	78 (100%)	78 (100%)	16 (100%)	16 (100%)
Spinal cord $D_{0.03}$< 54.3 Gy (RBE)	78 (100%)	78 (100%)	16 (100%)	16 (100%)

Abbreviations: sDLO: standard deep learning optimization, mDLO: manually adjusted deep learning optimization, CTV = clinical target volume, RBE = relative biological effectiveness, dose distribution, Voxmin = voxel-wise minimum dose distribution, voxmax = voxel-wise maximum dose distribution

Table S5 Target parameters, organ-of-interest doses and normal tissue complication probabilities of all patients (n=78). Average (min – max)

	**sDLO**	**mDLO**	**P_value**
Target parameters			
$CTV_{therapeutic}$ $V_{94\%}$ > 98 % voxmin	98.8 (97.4-99.7)	98.7 (97.5-99.6)	**<0.001**
$CTV_{therapeutic}$ D_1 < 78.0 Gy (RBE) voxmax	74.7 (73.3-76.4)	74.8 (73.4-76.2)	**0.003**
$CTV_{therapeutic}$ D_{mean}	70.3 (70.2-70.7)	70.3 (69.9-70.7)	0.159
$CTV_{elective}$ $V_{94\%}$> 98 % voxmin	98.4 (96.1-99.2)	98.4 (97.6-99.5)	0.572
**Serial OOIs**			
Brain D_3 < 63.1 Gy (RBE)	15.9 (0.1-39.2)	15.5 (0.1-40.6)	**0.005**
Brainstem $D_{0.03}$ < 63.1 Gy (RBE)	12.1 (0.2-27.9)	11.1 (0.2-25.5)	**<0.001**
Spinal cord D_0 <54.3 Gy (RBE)	28.6 (15.3-42.1)	29.1 (15.2-44.0)	**0.014**
Parallel OOIs			
D_{mean} Parotid Glands Gy (RBE)	14.6 (4.0-59.0)	14.5 (2.8-58.9)	**0.001**
D_{mean} Submandibular Glands Gy (RBE)	45.7 (10.8-70.0)	45.0 (11.0-70.0)	**<0.001**
D_{mean} Oral Cavity Gy (RBE)	25.9 (3.2-69.0)	25.6 (3.0-68.9)	**<0.001**
D_{mean} PCM Superior Gy (RBE)	46.4 (13.8-68.8)	46.0 (13.3-69.0)	**<0.001**
D_{mean} PCM Medius Gy (RBE)	39.0 (6.4-70.4)	38.3 (5.5-70.4)	**<0.001**
D_{mean} PCM Inferior Gy (RBE)	18.9 (8.0-57.6)	18.0 (6.5-57.4)	**<0.001**
D_{mean} Esophagus Gy (RBE)	11.1 (0.0-51.7)	9.7 (0.0-51.0)	**<0.001**
D_{mean} Thyroid Gy (RBE)	44.5 (16.1-53.5)	43.5 (15.6-53.3)	**<0.001**
NTCPs			
Grade ≥2 Dysphagia (%)	15.9 (2.9-64.6)	15.5 (2.8-65.2)	**<0.001**
Grade ≥3 Dysphagia (%)	3.8 (0.2-35.6)	3.7 (0.2-35.5)	**<0.001**
Grade ≥2 Xerostomia (%)	37.9 (24.0-70.7)	37.6 (22.8-70.2)	**<0.001**
Grade ≥3 Xerostomia (%)	10.6 (6.0-26.8)	10.5 (5.7-26.4)	**<0.001**

Min: minimum and max: maximum values are notes between parentheses.
Abbreviations: sDLO: standard deep learning optimization, mDLO: manually adjusted deep learning optimization, CTV = clinical target volume, OOIs = organs-of-interest, PCMs = pharyngeal constrictor muscles, RBE = relative biological effectiveness, dose distribution, Voxmin = voxel-wise minimum dose distribution, voxmax = voxel-wise maximum dose distribution, NTCPs = normal tissue complication probabilities. Significant differences between sDLO and mDLO (marked bold) with P <.013 for target parameters, P <.017 for serial OOIs, P <.006 for parallel OOIs, and P<.0125 for NTCPs

Table S6 Summary of predicted mean dose by an independent organ-of-interest doses prediction tool and mean doses of mDLO plans of all patients (n=78). Average (min – max)

OOIs	Predicted [Gy (RBE)]	mDLO [Gy(RBE)]	P value	Pearson R	Slope
Parotid left	16.2 (3.1-39.4)	14.1 (2.9-36.3)	<0.001	0.99	0.87
Parotid right	16.8 (5.8-59.6)	14.8 (5.4-58.9)	<0.001	0.99	0.9
Submandibular left	47.4 (16.9-67.2)	46.4 (15.2-70.0)	<0.001	0.99	0.98
Submandibular right	44.7 (13.6-67.3)	43.6 (11.0-69.8)	<0.001	0.99	0.98
Oral Cavity	26.9 (4.0-65.5)	25.6 (3.0-69.9)	<0.001	1.0	0.96
PCM Superior	47.1 (12.7-70.6)	46.0 (13.3-69.0)	<0.001	0.99	0.97
PCM Medius	40.5 (13.3-69.6)	38.3 (5.5-70.4)	<0.001	0.98	0.96
PCM Inferior	22.4 (10.0-61.2)	18.0 (6.5-57.5)	<0.001	0.96	0.83

Abbreviations: OOI: organ-of-interest, mDLO: manually adjusted deep learning optimization, PCM: pharynx constrictor muscle

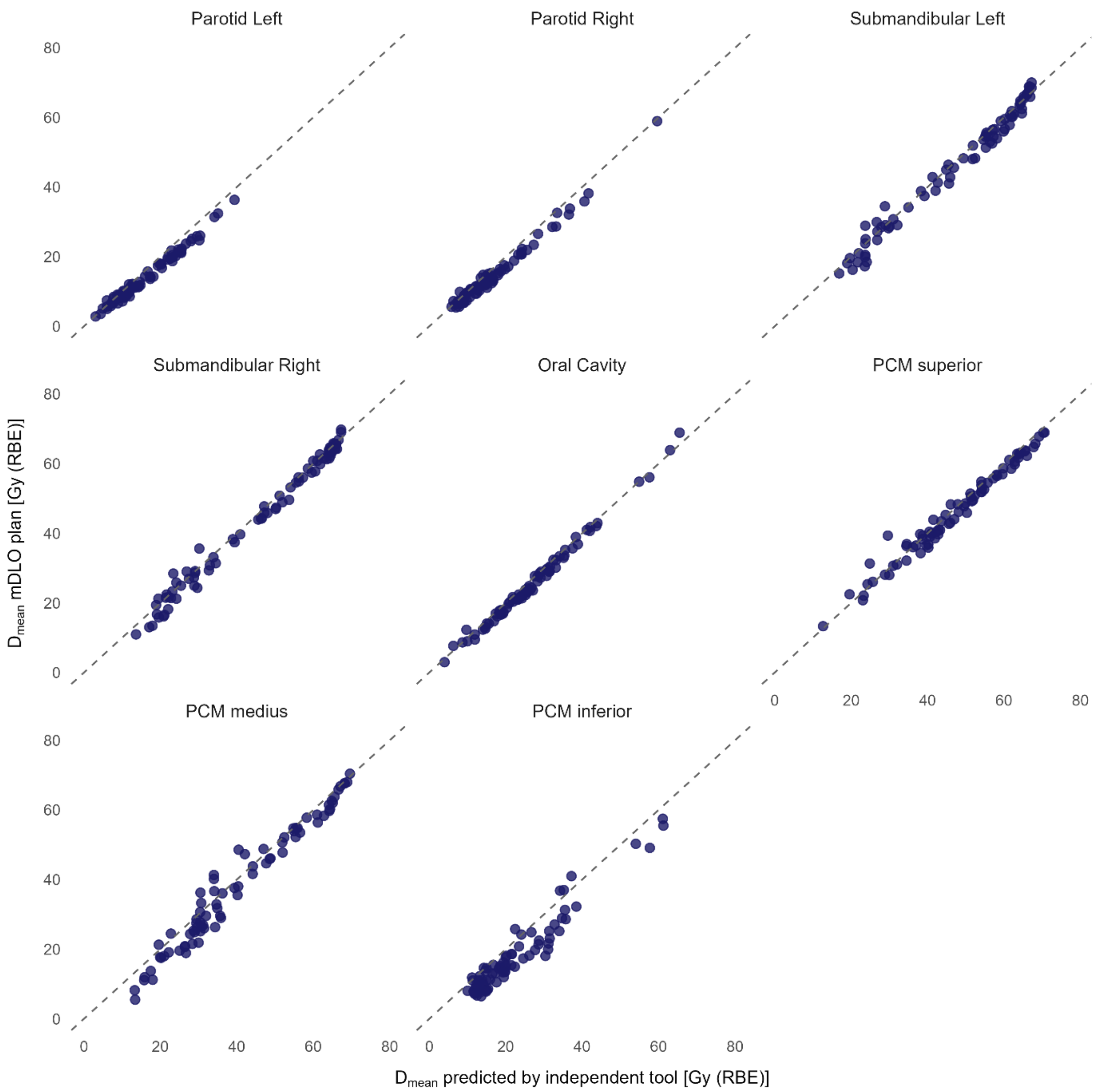

Fig. S1 Scatterplots showing the predicted mean dose (D_{mean}) versus the mean dose of the mDLO plan for eight organs-of-interest (OOIs). Each panel represents one OOI, which the diagonal dashed line indicating the identity line (y=x)

Table S7 Target parameters, organ-of-interest doses and normal tissue complication probabilities of mDLO and manual plans of benchmark cohort (n=16). Average (min – max)

	**mDLO**	**Manual**	**P_value**
Target parameters			
$CTV_{therapeutic}$ $V_{94\%}$ > 98% voxmin	98.7 (97.9-99.5)	98.6 (98.1-99.1)	0.464
$CTV_{therapeutic}$ D_1 < 78.0 Gy (RBE) voxmax	74.8 (74.0-76.2)	74.7 (73.3-75.7)	0.860
$CTV_{therapeutic}$ D_{mean}	70.3 (70.1-70.7)	70.3 (69.8-70.6)	0.289
$CTV_{elective}$ $V_{94\%}$ > 98% voxmin	98.4 (97.6-99.5)	98.4 (98.0-99.1)	0.860
Serial OOIs			
Brain D_3 < 63.1 Gy (RBE)	15.5 (0.1-34.4)	14.2 (0.1-28.9)	0.669
Brainstem $D_{0.03}$ < 63.1 Gy (RBE)	10.0 (0.2-23.2)	11.7 (0.2-22.7)	0.231
Spinal cord D_0 < 54.3 Gy (RBE)	30.3 (20.0-41.4)	33.2 (20.5-40.8)	**0.004**
Parallel OOIs			
D_{mean} Parotid glands	15.8 (5.6-38.2)	16.9 (5.6-40.1)	**0.002**
D_{mean} Submandibular glands	42.9 (13.0-70.0)	42.5 (14.5-70.1)	0.919
D_{mean} Oral Cavity	25.0 (9.0-54.8)	25.4 (8.9-54.9)	0.173
D_{mean} PCM Superior	43.3 (13.3-64.8)	43.7 (14.3-64.7)	0.706
D_{mean} PCM Medius	32.8 (13.8-68.0)	34.0 (13.4-67.5)	0.058
D_{mean} PCM Inferior	18.7 (6.7-55.5)	19.4 (7.1-57.4)	0.083
D_{mean} Esophagus	9.4 (0.6-28.9)	9.4 (1.1-27.9)	0.900
D_{mean} Thyroid	43.6 (30.2-50.7)	43.5 (30.9-50.0)	0.348
NTCPs			
Grade ≥2 Dysphagia	14.8 (2.8-65.2)	15.1 (2.9-65.3)	0.025
Grade ≥3 Dysphagia	5.2 (0.4-35.5)	5.4 (0.4-36.5)	0.025
Grade ≥2 Xerostomia	35.4 (25.3-49.1)	35.8 (26.3-50.3)	0.093
Grade ≥3 Xerostomia	9.5 (6.4-14.3)	9.6 (6.6-14.8)	0.105

Abbreviations and bonferroni-corrected p-values are similar as Supplementary Table S6

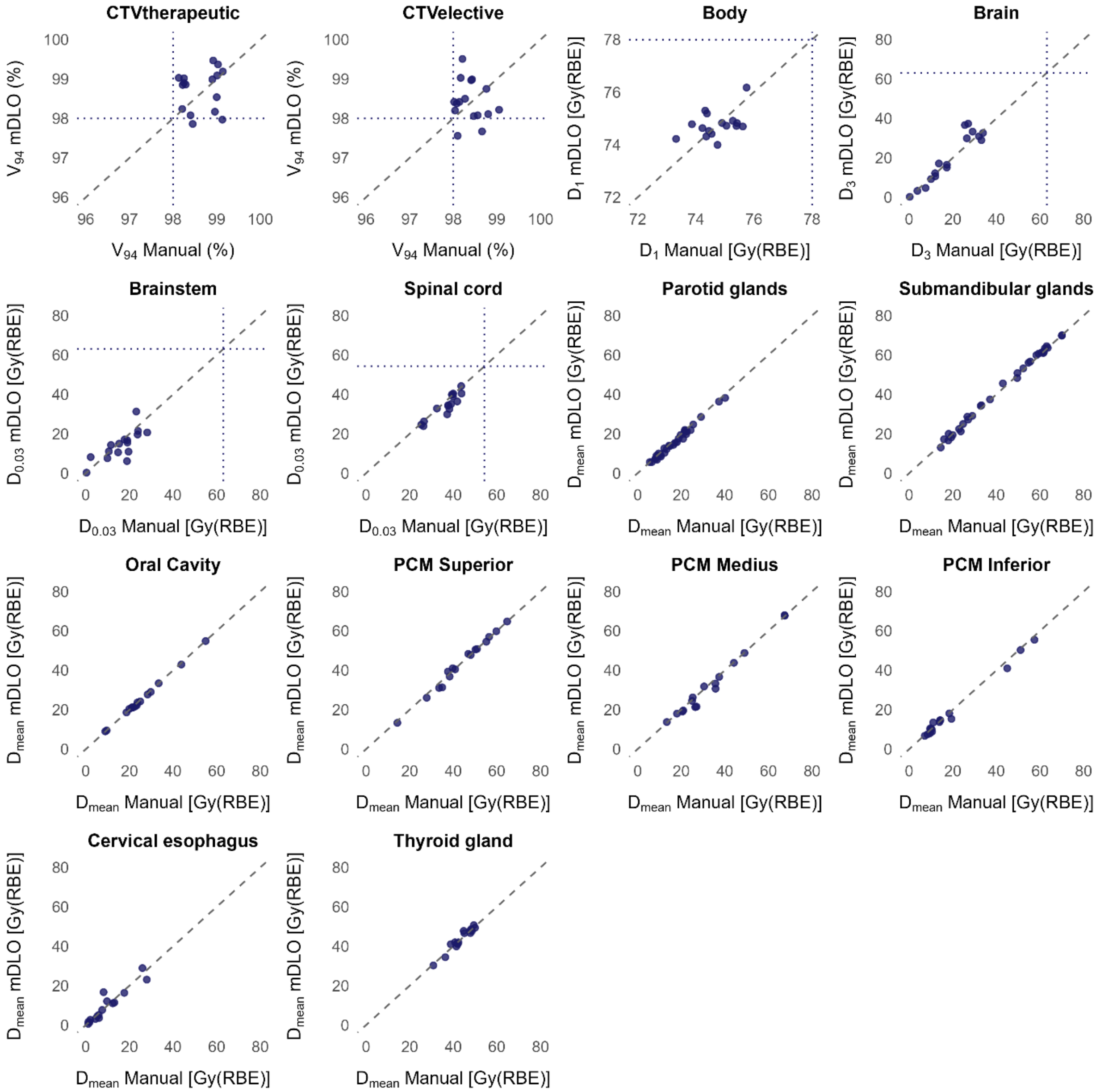

Fig. S2 Target parameters and organ-of-interest doses for mDLO and manual plans (n=16). The values are plotted as a blue dot for each patient, the dotted grey line represents the identity line (x=y). mDLO: manually adjusted deep learning optimization, RBE: relative biological effectiveness

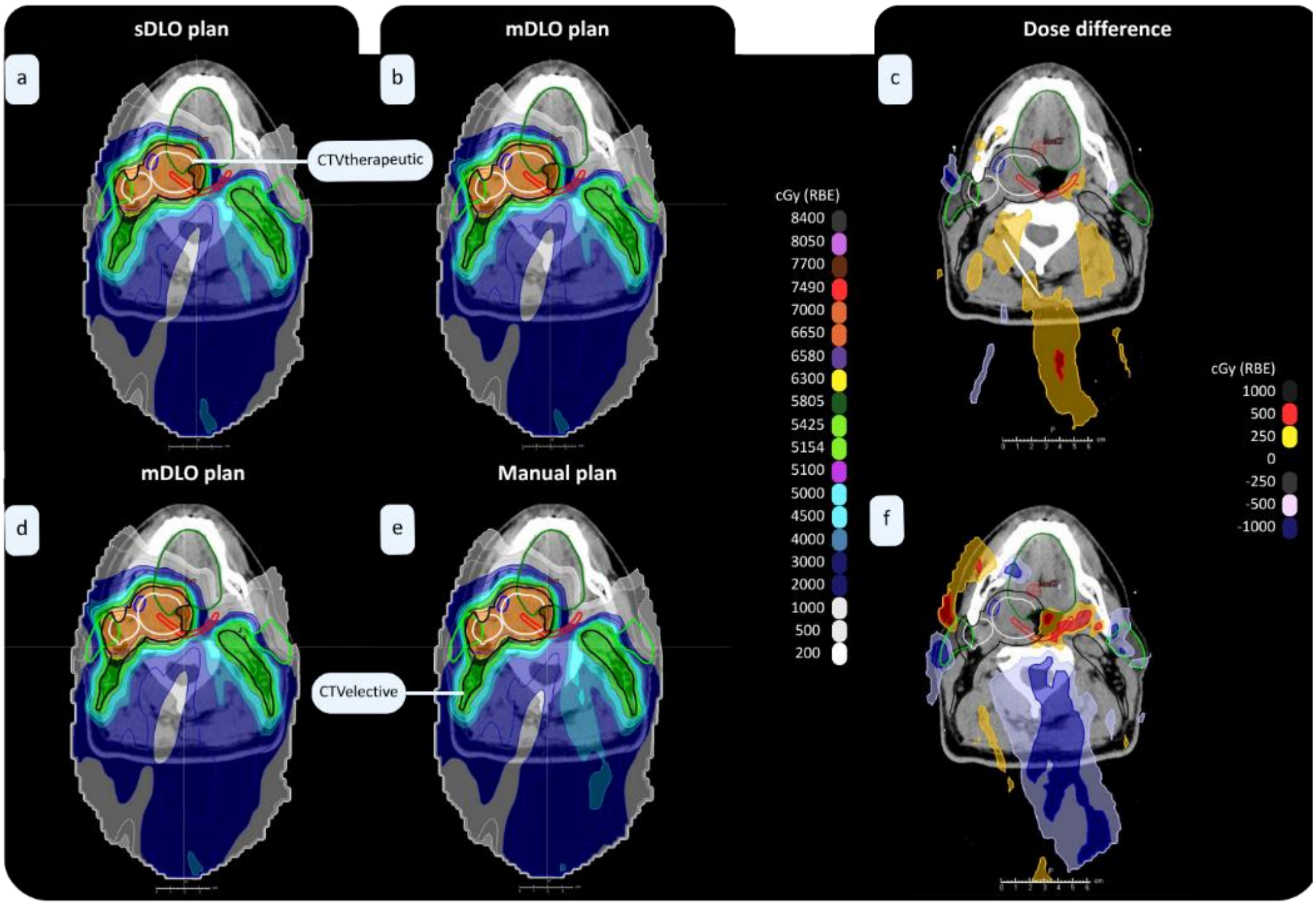

Fig. S3 A transversal cross-section of a sDLO (a), mDLO (b and d) and manual plan (e) of a representative case and the dose differences (sDLO minus mDLO, c) and (mDLO minus manual plan, f). Panels (b) and (d) include the same mDLO plan, presented for comparison with sDLO and manual plans, respectively. Oral cavity (green), pharynx constrictor muscle superior (red), parotid glands (green), submandibular gland (blue). Abbreviations: sDLO: standard deep learning optimization, mDLO: manually adjusted deep learning optimization, CTV: clinical target volume
